# ETS1, nucleolar and non-nucleolar TERT expression in nevus to melanoma progression

**DOI:** 10.18632/oncotarget.22254

**Published:** 2017-11-01

**Authors:** Jaskaren S. Kohli, Hira Mir, Afsheen Wasif, Heung Chong, Victoria Akhras, Rajiv Kumar, Eduardo Nagore, Dorothy C. Bennett

**Affiliations:** ^1^ Molecular and Clinical Sciences Research Institute, St George’s, University of London, London, UK; ^2^ Department of Cellular Pathology, St George’s University Hospitals NHS Foundation Trust, London, UK; ^3^ Department of Dermatology, St George’s University Hospitals NHS Foundation Trust, London, UK; ^4^ Division of Molecular Genetic Epidemiology, German Cancer Research Center, Heidelberg, Germany; ^5^ Department of Dermatology, Instituto Valenciano de Oncología, Valencia, Spain; ^6^ Current/Present address: European Research Institute for The Biology of Aging, University Medical Center Groningen, Groningen, The Netherlands; ^7^ Current/Present address: King’s College Hospital Foundation Trust, London, UK

**Keywords:** TERT, ETS1, melanoma, nevus, nucleolus

## Abstract

TERT (telomerase reverse transcriptase) is the catalytic component of telomerase. TERT shows little expression in normal somatic cells but is commonly re-expressed in cancers, facilitating immortalization. Recently-discovered *TERT* promoter mutations create binding sites for ETS-family transcription factors to upregulate TERT. ETS1 is reported to be important for TERT upregulation in melanoma. However it is unclear when in melanoma progression TERT and ETS1 proteins are expressed. To elucidate this question, ETS1 and TERT immunohistochemistry were performed on a panel of benign (n=27) and dysplastic nevi (n=34), radial growth phase (n=29), vertical growth phase (n=25) and metastatic melanomas (n=27). Lesions were scored by percentage of positive cells. ETS1 was readily detectable in all lesions, but not in normal melanocytes. TERT was located in either the nucleolus, the nucleoplasm (non-nucleolar) or both. Non-nucleolar TERT increased in prevalence with progression, from 19% of benign nevi to 78% of metastases. It did not however correlate with cell proliferation (Ki-67 immunostaining), nor differ significantly in prevalence between primary melanomas with or without a *TERT* promoter mutation. These results demonstrate that ETS1 is expressed very early in melanoma progression, and interestingly only non-nucleolar TERT correlates clearly in prevalence with melanoma progression. It can be acquired at various stages and by mechanisms other than promoter mutations.

## INTRODUCTION

One of the proposed hallmarks of cancer cells is immortality: the ability to proliferate indefinitely by bypassing cell senescence [1]. Cell senescence is a stable proliferative arrest following extensive cell division and telomere attrition, or other triggers including oncogene overexpression or genotoxic stresses [2]. Senescence can be triggered by short, dysfunctional telomeres, through DNA-damage signaling. In the germline and some stem cells, replicative senescence is prevented through telomere maintenance by active telomerase. TERT (telomerase reverse transcriptase), the catalytic component of telomerase, is transcriptionally repressed in normal adult somatic cells, which thus do senesce; but is reportedly re-expressed in 85-90% of human cancers, permitting immortality [3]. This re-expression seems to play a central role in carcinogenesis, making TERT and telomerase potentially important as drivers and therapeutic targets.

Benign and malignant primary pigmented lesions can be classified histologically into four main types with increasing malignancy, namely benign/banal melanocytic nevi, dysplastic nevi, thin, early melanomas termed radial growth phase (RGP) melanomas, and thicker, invasive or vertical growth phase (VGP) melanomas [4]. These types show progressively more genetic changes, disrupting characteristic molecular pathways, notably senescence, as reviewed in [5]. A benign nevus consists of a clonal mass of melanocytes that have senesced after proliferation triggered by an oncogenic mutation, usually in *BRAF* or *NRAS,* activating the MAPK pathway [6-10]. The cell-cycle inhibitor CDKN2A (isoform 1), hereafter called by its common name p16, appears the primary effector of senescence in melanocytes [11, 12], and is commonly expressed strongly in nevi [6, 7]. *CDKN2A* is a frequent site of mutations in familial melanoma [5-7]. Mutations or epigenetic disruption of the p16 pathway appear increasingly through the series, dysplastic nevi, RGP and VGP melanoma [7, 9]. VGP melanomas also tend to accumulate mutations suppressing apoptosis, discussed elsewhere [13, 5]. p16 deficiency contributes to senescence bypass, but telomere extension is also essential. Functional tests indicate that immortality emerges only late in melanoma progression [13]. Average telomerase activity rises strongly in later progression [14, 15]. Much interest was generated by recent findings that this rise is commonly mediated through mutations at hotspots in the *TERT* promoter, creating binding sites for ETS-family transcription factors [16-18] and facilitating *TERT* transcription [19]. Such mutations appear much more common in metastatic than primary melanomas [16, 20], yet are also detected in some early lesions intermediate between nevi and melanomas [9], and also (rarely) in the germline of some melanoma-prone families [16, 18].

Little is understood of TERT protein expression in relation to these mutations and to progression. Availability of a reliable anti-TERT antibody [21] has enabled us to investigate this by TERT immunohistochemistry (IHC) in nevi and melanomas. ETS1 expression was also re-examined. ETS1 has been implicated as a predominant factor activating mutant *TERT* promoters in melanoma [19], but previous studies have reached differing conclusions on ETS1 expression relative to melanoma progression [22-24].

## RESULTS

### Role of antigen-retrieval pH; revisiting p16 expression in melanoma progression

Antigen retrieval for IHC is commonly done at pH 6. However optimization tests showed strongest immunostaining after retrieval at pH 9, for all antibodies used here (Supplementary Figures 1-4). Retrieval at pH 9 was therefore used throughout this study.

For comparison with TERT expression, we re-examined p16 immunostaining, also to test whether the strong staining would increase apparent prevalence, as p16 is detected heterogeneously in nevi [6, 7]. However, while more cells were positive, the results (Supplementary Figure 5) were comparable to those reported before [25, 27-29], strengthening evidence that p16 expression is heterogeneous in many nevi and absent from most VGP melanomas.

### ETS1 expression with melanoma progression

**Figure 1:**
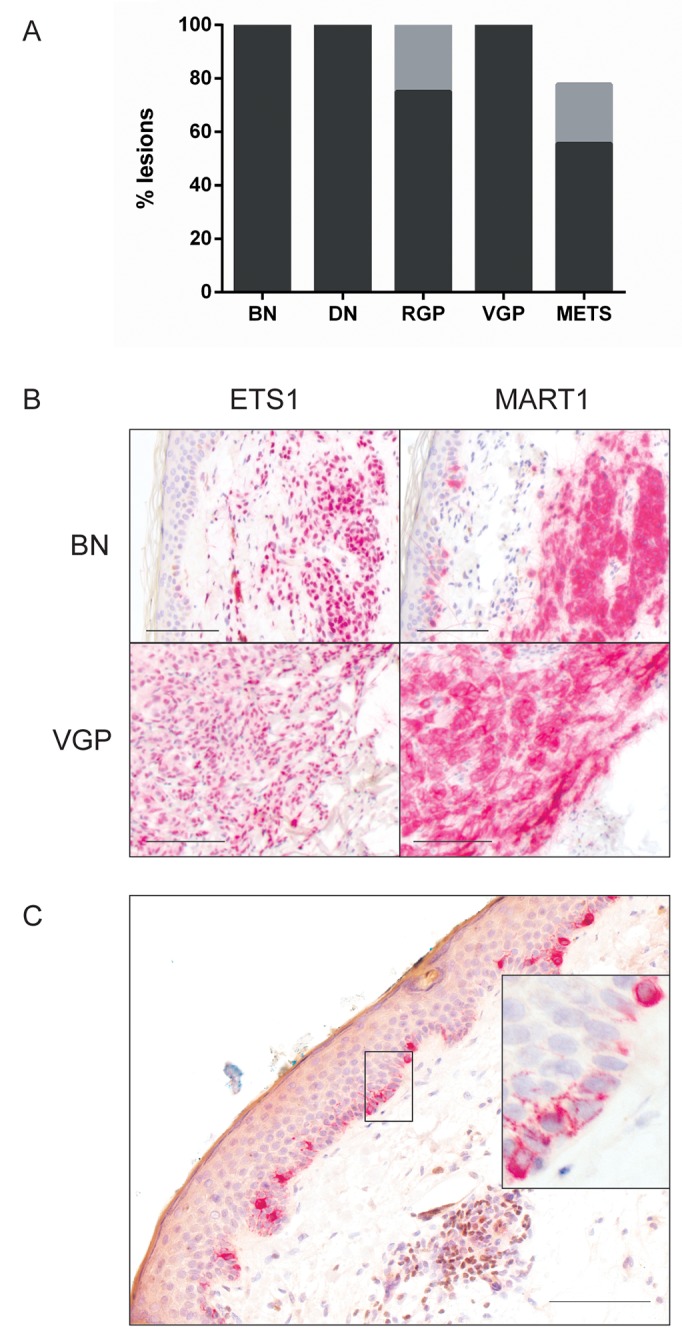
ETS1 expression with melanoma progression **(A)** Percentage of lesions showing high (>50% cells) (black) and moderate (5-50%) prevalence (grey). Prevalence <5% was not reproducibly distinguished from 0 by independent observers and thus had uncertain significance [25]; not shown. BN: benign nevus (n=10); DN: dysplastic nevus (n=10); RGP (n=8); VGP (n=9); METS: metastatic melanoma samples (n=9). **(B)** Representative highly prevalent ETS1 immunostaining in a benign nevus and VGP melanoma, with parallel MART1 IHC to show position of lesional cells. **(C)** ETS1 and MART1 double IHC in normal skin. Epidermal normal melanocytes express cytoplasmic MART1 (red) but no nuclear ETS1 (brown); expanded in inset. A group of lymphoid cells in the dermis (brown nuclei) provides a positive ETS1 control. Scale bars: 100 μm.

Nuclear ETS1 was expressed extensively and strongly in all lesions at all five tested stages of progression, except for a few metastases (Figure 1A, 1B). To compare normal melanocytes *in vivo* we examined normal epidermis in benign nevus sections. No ETS1 was detected. To test normal melanocytes specifically, double immunohistochemistry was performed on normal human skin for ETS1 and the melanosomal protein MART1 (MLANA) (Figure 1C). Epidermal melanocytes, identified by cytoplasmic MART1, were confirmed negative for nuclear ETS1. Thus, in melanocytes *in vivo*, ETS1 expression appears to be activated upon nevogenesis.

### TERT expression with melanoma progression: nucleolar and non-nucleolar

**Figure 2:**
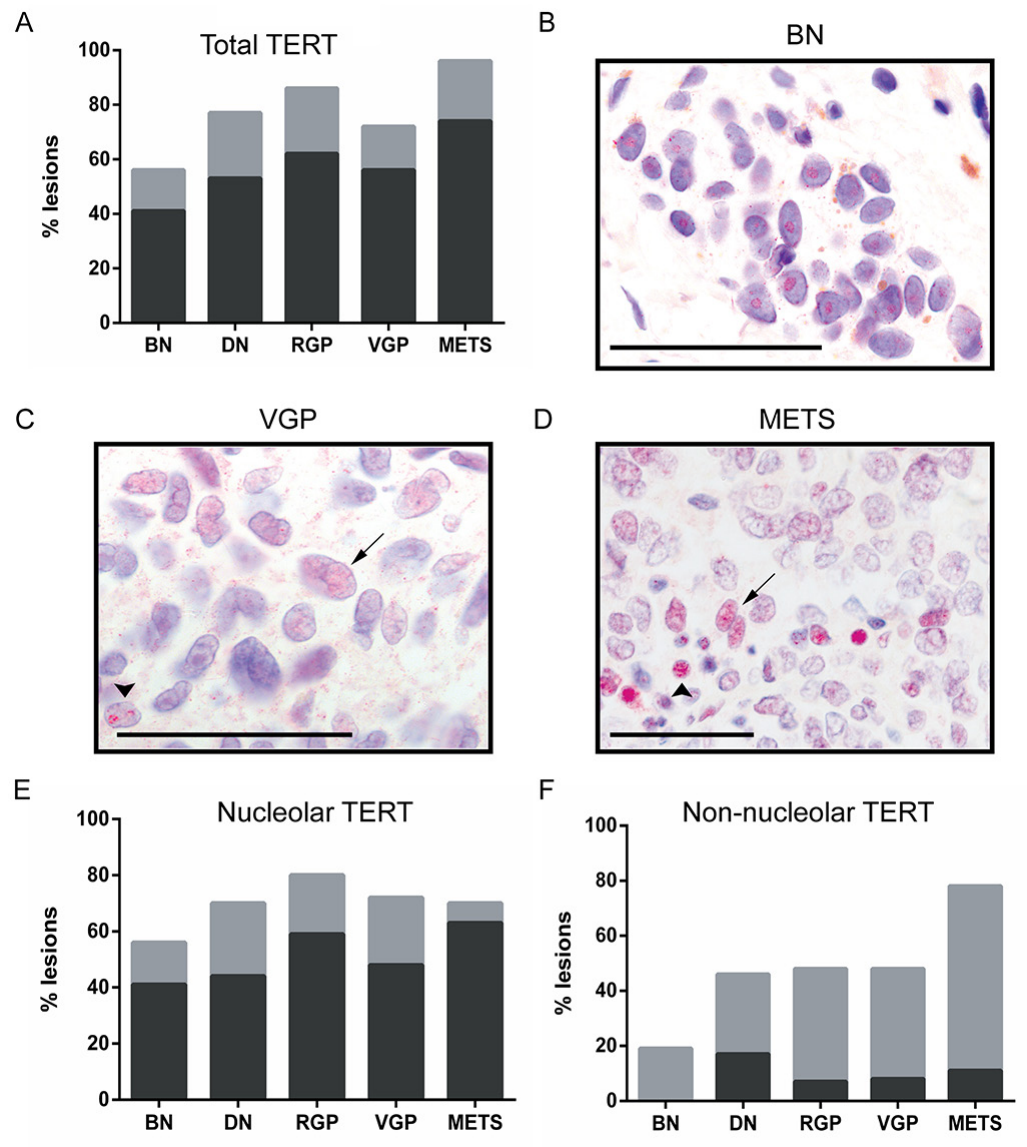
TERT expression and localization with melanoma progression Abbreviations and bar color coding as in Figure 1. BN (n=27), DN (n=34), RGP (n=29), VGP (n=25) and METS (n=27). **(A)** Total TERT prevalence, showing a borderline trend with progression (p=0.054). **(B)** Nucleolar TERT in a benign nevus nest (brown granules are melanin). **(C, D)** Non-nucleolar TERT in melanomas, diffuse in nucleoplasm (arrows) or in punctate nuclear foci (arrowheads). Scale bars: 50 μm. **(E)** Nucleolar TERT prevalence, showing no trend with progression (p=0.41). **(F)** Non-nucleolar TERT prevalence, showing a highly significant rise with progression (p=0.006). Significance testing was by χ^2^ tests.

TERT levels in the various lesional types are shown in Figure 2A. There was a numerical positive trend in total TERT prevalence, but with borderline statistical significance (p=0.0545). Benign and dysplastic nevi often displayed surprisingly widespread TERT positivity.

However, it was noticed that subcellular TERT location varied between lesions. In benign nevi TERT was typically localized solely to the nucleolus (Figure 2B). However in melanomas the protein was often found in distinct subnuclear foci, or diffusely across the nucleus (Figure 2C, 2D), designated ‘non-nucleolar’. Lesions were then recounted, discriminating nucleolar and non-nucleolar TERT (Figure 2E, 2F). There was no trend for nucleolar expression (p=0.41), but a statistically highly significant positive trend for non-nucleolar TERT detection with melanoma progression (p=0.006).

Non-nucleolar TERT was typically present heterogeneously, in 5-50% of cells within a lesion (grey areas, Figure 2F). It was found in less than 20% of benign nevi, in 40-50% of dysplastic nevi and primary melanomas, but in nearly 80% of metastatic samples. Prevalence differed significantly between benign nevi and all other lesions combined (p=0.004); also between metastatic melanomas and all other lesions combined (p=0.001). Taken together, these results suggest that gain of non-nucleolar TERT occurs in a few benign nevi, in some dysplastic nevi, but most commonly at the transition to metastasis.

To ensure specificity, TERT peptide blocking was performed, on two lesions with exclusively nucleolar (Supplementary Figure 6A), and predominantly non-nucleolar immunostaining respectively (Supplementary Figure 6B). Nuclear TERT detection was greatly reduced or absent in both cases, supporting high antibody specificity and that the antigen in both subnuclear locations is indeed TERT.

### Non-nucleolar TERT does not associate with cellular proliferation

Hypothesizing that non-nucleolar TERT might be linked to immortalization, we tested whether its presence correlated with proliferation, by immunostaining adjacent sections for proliferative marker Ki-67.

**Figure 3:**
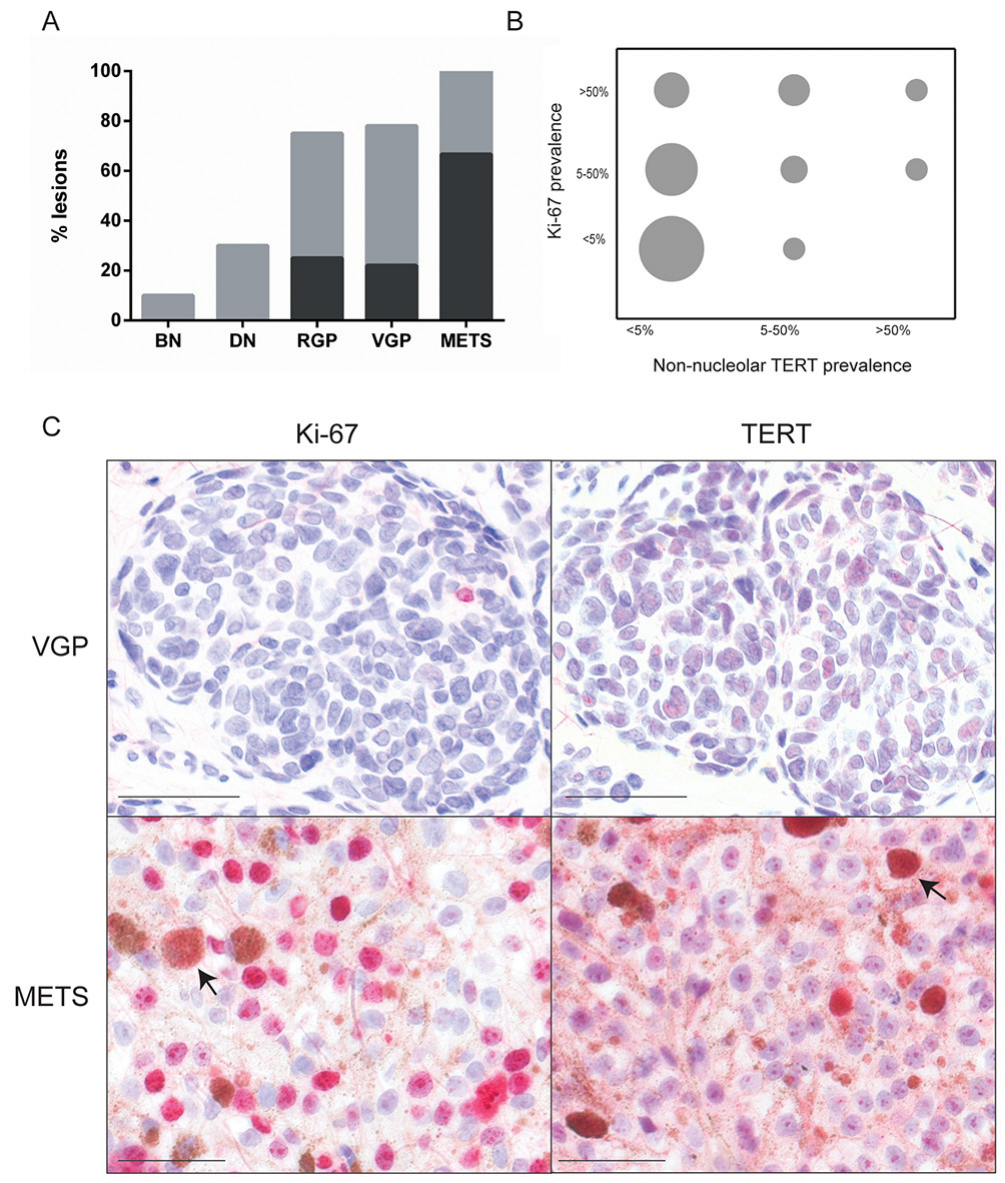
Ki-67 expression and lack of association with non-nucleolar TERT **(A)** Ki-67 prevalence, highly significantly rising with melanoma progression (p<0.0001, χ^2^ test). Abbreviations and bar color coding as in Figure 1. BN (n=10), DN (n=10), RGP (n=8), VGP (n=9) and METS (n=10). **(B)** Grouped scatter plot illustrating poor agreement between Ki-67 and non-nucleolar TERT prevalence (κ=0.15). The area of each circle is proportional to the number of lesions with the indicated prevalence for each marker. **(C)** Examples of lesions displaying poor agreement. Top: highly prevalent TERT but no Ki-67 expression. Bottom: widespread Ki-67 but very little non-nucleolar (although much nucleolar) TERT expression. Arrows: melanophages (macrophages which have engulfed melanin). Scale bars: 50 μm.

Ki-67 expression was well correlated with melanoma progression (p<0.0001), as expected [30]. Expression was rare in nevi but common in primary melanomas; nearly all metastases were >50% positive (Figure 3A). There was poor statistical agreement however between Ki-67 and non-nucleolar TERT in individual lesions (Figure 3B; κ=0.15). Examples of divergence between Ki-67 and TERT are shown in Figure 3C. Still, all lesions with >50% cells positive for non-nucleolar TERT, and most of those with 5-50%, were also Ki-67 positive. The lack of correlation was attributable to frequent lesions with Ki-67 but no/rare (<5%) non-nucleolar TERT.

### Non-nucleolar TERT expression does not associate with *TERT* promoter mutation status in primary melanomas

Since prevalence of both non-nucleolar TERT and TERT promoter mutations correlate with melanoma progression, we investigated whether non-nucleolar TERT might be a marker for these mutations. Primary melanomas with known *TERT* mutation status were now also analyzed by TERT IHC.

**Figure 4:**
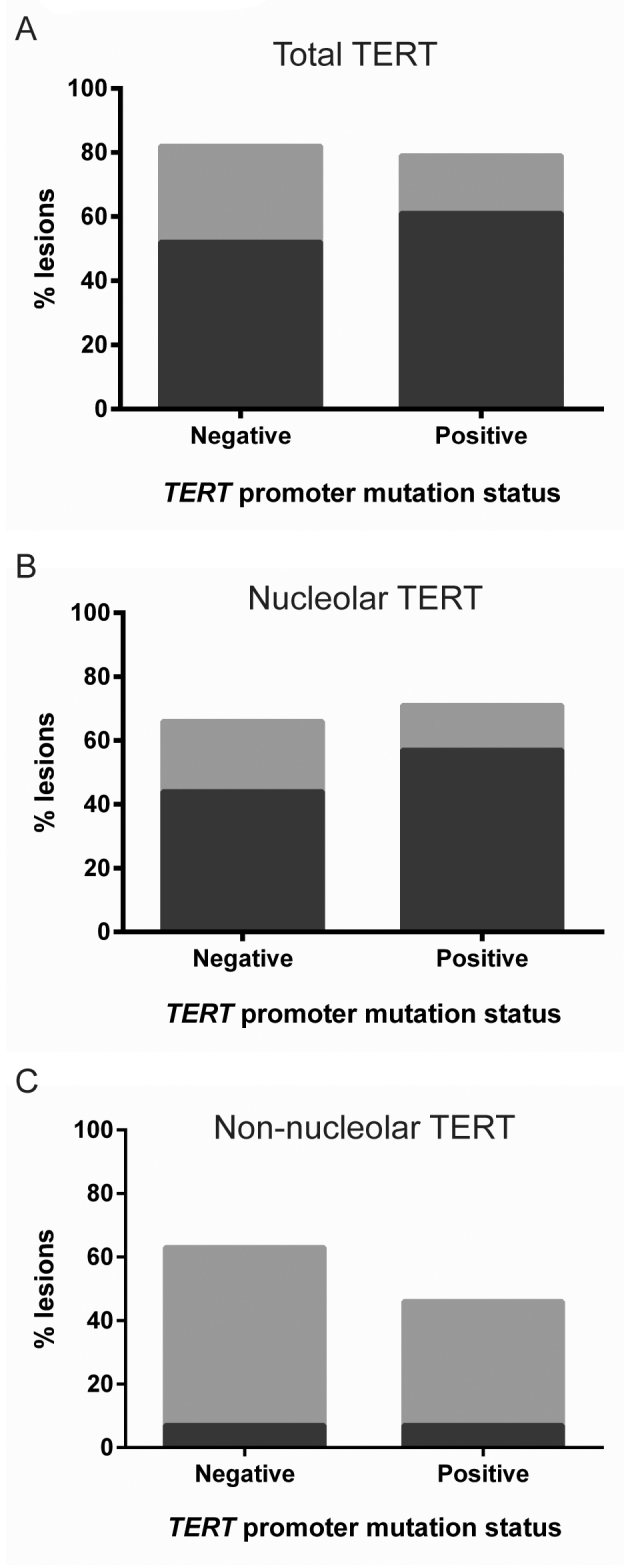
TERT expression in primary melanomas with and without a *TERT* promoter mutation No significant difference was observed between primary melanomas with (n=27) or without a *TERT* promoter mutation (n=28), for **(A)** total TERT (p=0.59), **(B)** nucleolar TERT (p=0.62) or **(C)** non-nucleolar TERT (p=0.45), by χ^2^ tests. Bar color coding as in Figure 1.

No significant differences were found however between melanomas with or without a *TERT* promoter mutation, in either total (p=0.59, Figure 4A), nucleolar (p=0.60, Figure 4B), or non-nucleolar TERT detection (p=0.45, Figure 4C). These results indicate that TERT can also be upregulated in melanoma through mechanisms other than promoter mutations.

### Nucleolar TERT location in cultured melanocytic cells

We next investigated TERT via immunocytochemistry in melanocytic cell cultures, to compare normal and malignant cells and test the hypothesis that non-nucleolar TERT might indicate cell immortality. We used A375P melanoma cells, Hermes 3A TERT/CDK4-immortalized human melanocytes [7], and SGML-2 normal human melanocytes. Cells were also immunostained for nucleolar marker nucleolin, and for coilin, a marker of Cajal bodies, a reported site of telomerase assembly [31].

**Figure 5:**
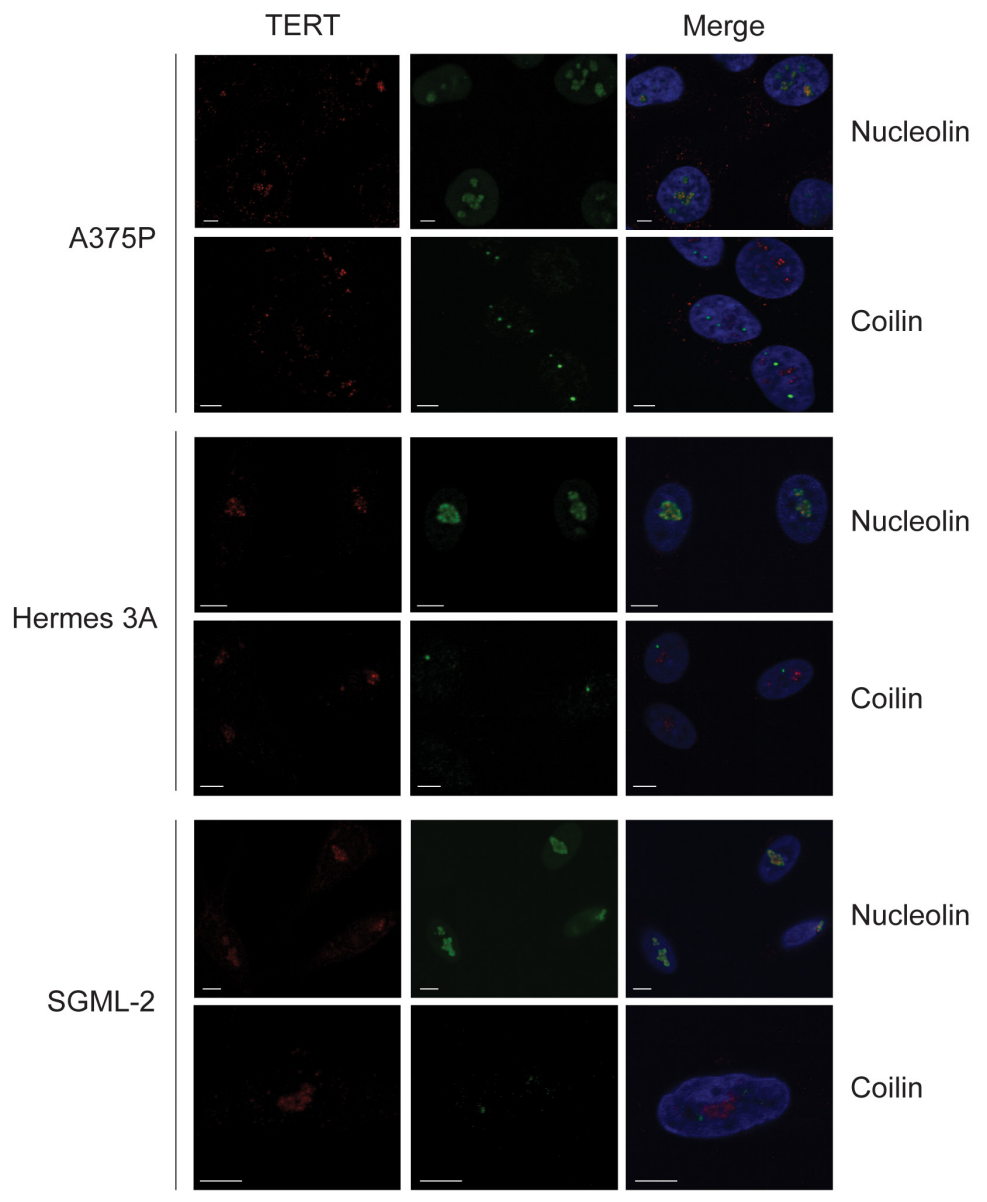
TERT colocalization with nucleolin, not coilin, in cultured human melanoma cells and melanocytes A375P is an immortal melanoma line, Hermes 3A cells are immortalized melanocytes and SGML-2 is a diploid adult melanocyte strain. All cells were proliferating. Panels show double immunofluorescence for TERT (red) with nucleolin or Cajal body marker coilin (green). Nuclei visualized with DAPI (blue). Scale bars: 5 μm. TERT localized to nucleoli, not Cajal bodies, in all cells in these cultures.

Interestingly, in all three cell lines TERT was present and localized entirely to nucleoli (Figure 5). No co-localization was observed with coilin, indicating TERT is not (or only very transiently) found in Cajal bodies in these cells. The finding also seemed inconsistent with non-nucleolar TERT as an indicator of immortality. We tested whether the nucleolar location here might be an artefact of fixation, given TERT’s high isoelectric point (∼11.3) [32] and hence positive charge at near-neutral pH. The nucleolus (rich in negatively charged RNA) has been reported to accumulate positively charged proteins [33]. Cells were therefore now fixed at pH 10 to neutralize TERT. No change in TERT location was observed however (Supplementary Figure 7), suggesting it was not artefactual. The expression of TERT in non-immortal melanocytes in culture but not *in vivo* remains unexplained, and these data indicate no simple link between non-nucleolar TERT and immortality or active telomerase.

## DISCUSSION

ETS1 expression was detected in all nevi and but not in normal epidermal melanocytes, agreeing with [24] but not with [23]. This upregulation may occur because nevi are senescent; ETS1 upregulation was reported in senescent fibroblasts [34]. It may also be because nevi carry an activated oncogene, activating ETS1 by phosphorylation downstream of RAS/RAF/MAPK signaling [5], and possibly increasing its abundance. At any rate, this presence of ETS1 could allow TERT expression in a nevus cell acquiring an oncogenic *TERT* promoter mutation, since these mutations reportedly associate with increased TERT transcription and protein expression, in the presence of ETS-family factors [20, 35]. This would activate telomerase, potentially impairing senescence stability and being clonally selective.

TERT immunostaining has not to our knowledge previously been analyzed as nucleolar or non-nucleolar relative to melanoma progression. We found that only non-nucleolar TERT increased significantly in prevalence with melanoma progression. Benign nevi had predominantly nucleolar TERT, aligning with a report that nucleolar TERT is not functional [36]. However the “melanoma-like” non-nucleolar TERT was also found in 20% of benign nevi, and 40% of dysplastic nevi. *TERT* promoter mutations have also been detected as an early event in some “intermediate” (dysplastic-like) lesions [9], indicating remarkably that active telomerase may be selective even within a largely senescent tissue. Dysplastic nevi were estimated to transform very rarely into melanoma (∼1 in 30,000) [37], implying that the non-nucleolar TERT seen here in about 40% of dysplastic lesions is not sufficient for immortality. This conforms with the finding that some *p16* mutations apparently arise only after *TERT* promoter mutations [9]. One wonders how such TERT mutations can become clonally expanded, in a nevus with unmutated p16. Perhaps in these cases one or both p16 copies are silenced by methylation, or TERT allows some repair of telomeric DNA damage even during arrest, and this reduces p16 activation.

No significant difference in TERT protein expression was observed between primary melanomas with or without a mutated *TERT* promoter; thus IHC cannot be used as an assay for the presence of the mutation, as has been hoped. This initially seemed surprising, but findings were similar in recent studies of glioblastoma [38] and a previous report on melanoma [39] (although without stated validation of their antibody, which gave strong cytoplasmic staining). Evidently *TERT* expression can be activated by mechanisms other than mutation. For example, Fan et al [40] reported a positive correlation between multiple cytosine methylation in part of the *TERT* promoter (667-482 bp upstream of the ATG start site) and TERT expression, in melanomas arising from congenital nevi or lacking a TERT promoter mutation. They cited similar previous findings in brain tumors. Conversely, the -146 C>T *TERT* promoter mutation has been reported to require non-canonical NF- κB signaling to co-operate with ETS1 [41]. Thus lesions carrying this mutation but negative for TERT protein may lack this signaling, or another co-operating factor. Moreover it was recently reported that these promoter mutations alone lead to low levels of TERT mRNA expression, below detection limits and insufficient for maintenance of all telomeres, so that additional changes seem to be needed for further upregulation and cell immortalization [42], consistent with the present data.

Nucleolar TERT expression did not alter with melanoma progression. The nucleolus was reported as a site of telomerase assembly, from where it would relocate to telomeres during DNA replication [31, 43]. This might predict a correlation of non-nucleolar TERT immunostaining with cell proliferation (Ki-67 prevalence), which we therefore tested for, but did not see, suggesting additional reasons for extranucleolar location. Others have suggested that the nucleolus regulates telomerase activity. Transfection of exogenous *TERT* into various malignant cells gave a pan-nuclear localization (i.e. including non-nucleolar), but in normal diploid fibroblasts it localized solely to the nucleolus [36, 44-46]. These results suggest dysregulation of TERT localization in malignant cells. Nucleolin and PINX1 are reportedly both required for localizing TERT to the nucleolus [44, 46]. We reported increasing nucleolin expression with melanoma progression [25], which does not readily explain the trend for TERT; however we know of no data on PINX1 expression in melanoma.

In summary, we report strong ETS1 expression in all nevi and melanomas but not normal melanocytes, providing a mechanism for how early TERT promoter mutations can be selective following oncogene activation. We also report for the first time a pathologically significant aspect of TERT localization relative to the nucleolus: only non-nucleolar TERT increases in prevalence with melanoma progression. It will be important to investigate in future the mechanism and significance of this non-nucleolar location, which did not seem to be linked directly to cellular immortality, at least in our cell cultures. No significant difference in TERT expression or location was observed between primary melanomas with or without a *TERT* promoter mutation, consistent with the conclusion that *TERT* upregulation is more important for the development of a melanoma than the mechanism of upregulation.

## MATERIALS AND METHODS

### Patients and specimens

This study was approved by the West London and GTAC Research Ethics Committee (ref. 08/H0803/154). Samples were obtained from patients at St George’s Hospital, with written consent. A total of 142 lesional samples were used for TERT IHC: 27 benign nevi, 34 dysplastic nevi, 29 RGP melanomas, 25 VGP melanomas and 27 metastatic melanomas. Smaller numbers of lesions were available for IHC of other proteins (see figure legends). Blinded samples of primary melanomas with a wild-type (n=28) or mutated *TERT* promoter (n=27) were sent to London from the Biobank of the Instituto Valenciano de Oncología. Samples were obtained from patients at the Institute with written consent.

### Immunohistochemistry

Detailed protocols for immunohistochemistry and peptide-blocking are given in the Supplementary Materials. Sections were examined by microscope and classed as containing either <5%, 5-50% or >50% positive lesional cells, a system giving high agreement between two independent scorers [25]. All specimens were evaluated by two scorers. Where scores did differ, lesions were re-examined jointly to reach agreement. Statistical analyses, using GraphPad Prism version 6.07, were χ^2^ tests for significance of differences between categories and a kappa test for concordant immunostaining of two proteins. *p*<0.05 was considered significant.

### Cell cultures

A375P melanoma cells and Hermes 3A immortal human melanocytes were from the Wellcome Trust Functional Genomics Cell Bank at St George’s. Cell Bank cultures are validated by IDEXX Bioresearch and were grown by us for less than 6 months from recovery of cryopreserved stocks. SGML-2 was a normal adult epidermal melanocyte strain explanted at St George’s from an abdominoplasty; cells were cultured as described [26].

### Double immunofluorescence

Cells were grown on glass coverslips in 16-mm wells, washed twice in PBSD (complete Dulbecco’s PBS with CaCl_2_ and MgCl_2_) and fixed in 4% paraformaldehyde in PBSD for 20 minutes. After two washes in PBSD, cells were permeabilized with 0.1% Triton X-100 in PBSD for 4 minutes. Three washes in PBSD were carried out before blocking in 10 mg/ml BSA in PBSD. Cells were left overnight in primary antibody diluted in 10 mg/ml BSA in PBSD, at 4°C in a humidified chamber. Primary antibodies used were against TERT (EST21-A, Alpha Diagnostic International, 1:500), coilin (ab87913, Abcam, 1:1000) and nucleolin (ab136649, Abcam, 1:2000). After five brief washes with PBSD, cells were incubated in Alexa Fluor 594-conjugated goat anti-rabbit IgG (A-11012, Life Technologies, 1:400) for detection of TERT and Alexa Fluor 488-conjugated goat anti-mouse IgG (A-11008, Life Technologies, 1:400) for nucleolin or coilin. After 3 washes in PBSD, cells were counterstained with 1 μg/ml DAPI for 10 min, washed 3x in PBSD and once in dH_2_O, then mounted on slides in Citifluor. A LSM 510 Zeiss confocal microscope and ZEN 2009 software (Zeiss) were used to capture images.

## SUPPLEMENTARY MATERIALS FIGURES


