# Supplementary material for: ETS1, nucleolar and non-nucleolar TERT expression in nevus to melanoma progression
Source: Oncotarget. 2017 Nov 1;8(61):104408–17. doi: 10.18632/oncotarget.22254 (PMC5732815; doi:10.18632/oncotarget.22254)
Supplement: Supplementary file 1 [file oncotarget-08-104408-s001.pdf]

ETS1, nucleolar and non-nucleolar TERT expression in nevus to melanoma progression

SUPPLEMENTARY MATERIALS

Immunohistochemistry

All reagents used in IHC were purchased from Leica Biosystems unless stated. A volume of 150 µl was used for each step. IHC was carried out with the Leica Bond III automated system. 4 µm formalin fixed, paraffin embedded tissue sections were placed on to X-tra adhesive slides. They were then deparaffinized in Bond Dewax solution for 30 minutes at 72°C and then washed three times in 100% alcohol and then three times in wash buffer. Two washes in epitope retrieval solution 1 (citrate based at pH 6) or epitope retrieval solution 2 (EDTA based at pH 9) were then carried out. Antigen retrieval was carried out by heating sections in one of these retrieval solutions at 100°C for 30 minutes. Another wash in the same retrieval solution and then three washes in wash buffer were carried out. Sections were then incubated in primary antibody, diluted in antibody diluent, for 15 minutes. Primary antibodies used in this study were TERT (EST21-A, Alpha Diagnostic International, 1:50), ETS1 (NCL-ETS-1, Novocastra, 1:25), MART1 (NCL-L-MelanA, Novocastra, 1:25), Ki-67 (ab16667, Abcam, 1:100) and p16 (sc-65476, Santa Cruz Biotechnology, 1:100). Two washes in wash buffer were carried out before a post-primary rabbit anti-mouse IgG was added for 20 minutes. Another two washes occurred in wash buffer for 2 minutes each before an alkaline phosphatase conjugated anti-rabbit IgG was added for 30 minutes (this conjugated antibody acts as a secondary antibody to rabbit primaries and tertiary antibody to mouse primaries). Two washes in wash buffer for 2 minutes each, another wash in wash buffer for 5 minutes and then a brief wash in dH_2O were carried out. Fast Red chromogen was added for 10 minutes, removed, and then fresh chromogen was added for a further 5 minutes. Sections were washed in dH_2O before nuclei were counterstained in hematoxylin for 5 minutes. Final washes in water, wash buffer and water again were carried out successively.

For double immunohistochemistry, the protocol was carried out as mentioned but with minor adjustments. After the first primary antibody incubation, 3% hydrogen peroxide was added to block endogenous peroxidase activity. This was required as an anti-rabbit IgG conjugated to horseradish peroxidase was used and 3'3-diaminobenzidine replaced Fast Red. Before counterstaining in hematoxylin, the second primary antibody was added and the process repeated with the alkaline phosphatase conjugated antibody and Fast Red chromogen.

Peptide blocking against the TERT antibody was carried out by incubating 1 µg of antibody with 5 µg, 10 µg or 15 µg of the peptide corresponding to the epitope of TERT used to raise the antibody (peptide from Alpha Diagnostic International), in a final volume of 500 µl PBS (Invitrogen) overnight at 4°C. Immune complexes were pelleted out by centrifuging the mixture at 13,000 rpm for 15 minutes the next day. The supernatant was aspirated and used as a primary antibody substitute using the protocol above.

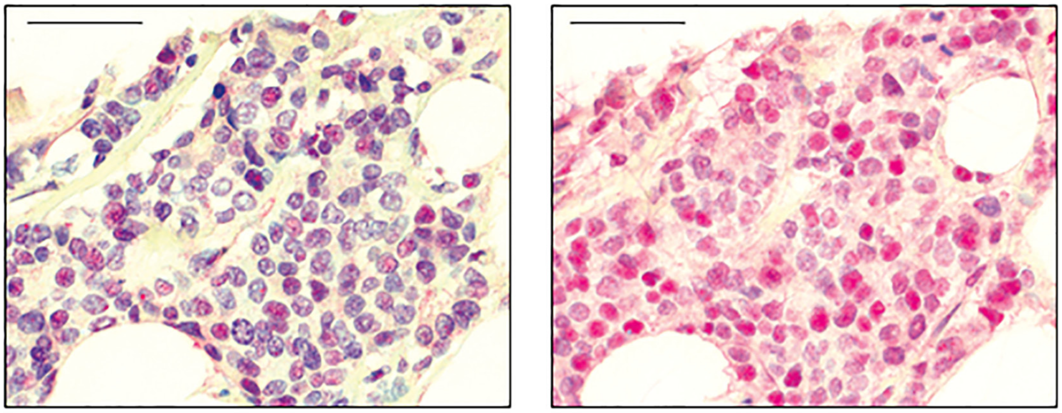

Supplementary Figure 1: IHC against TERT in a breast carcinoma using an acidic or basic antigen retrieval system. TERT immunostaining is more intense and there are more positive cells after antigen retrieval at pH 9 (right) than at pH 6 (left). Note that non-nucleolar TERT immunostaining is seen in this malignancy as well. Scale bars = 50 µm.

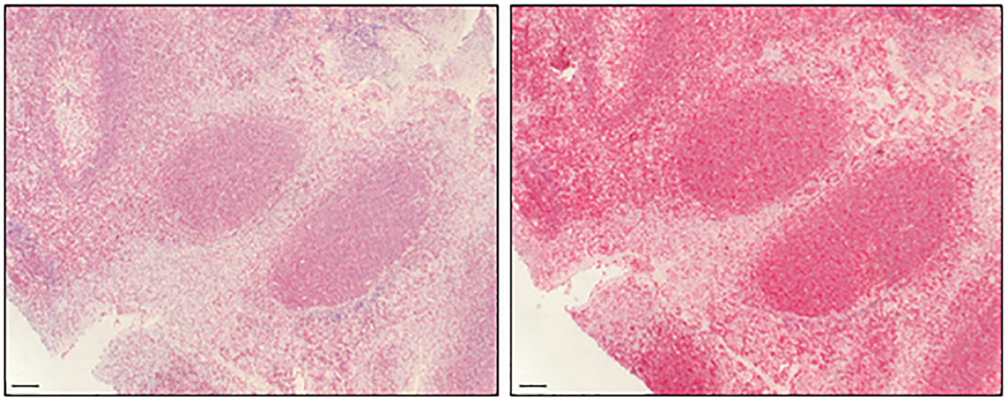

Supplementary Figure 2: IHC against ETS1 in tonsil tissue (lymphoid cells) using acidic or basic antigen retrieval. ETS1 immunostaining is clearly more intense after retrieval at pH 9 (right) than at pH 6 (left). Scale bars = 100 µm.

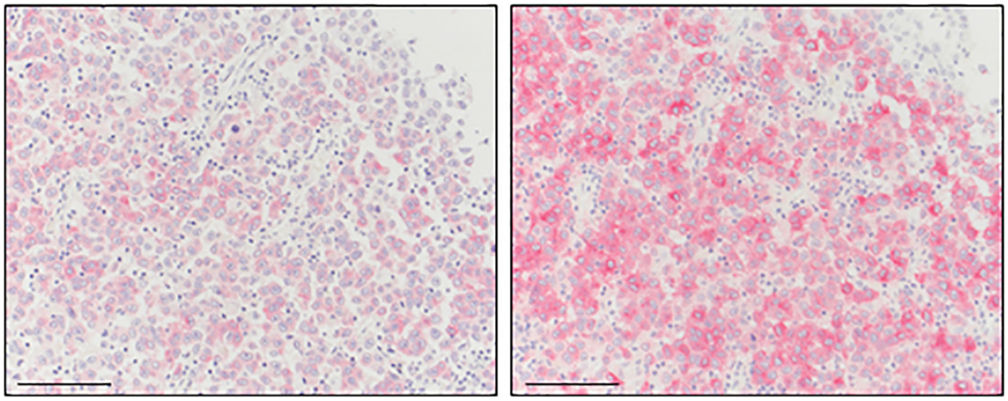

Supplementary Figure 3: IHC against MART1 in a melanoma using acidic or basic antigen retrieval. MART1 immunostaining is clearly more intense after retrieval at pH 9 (right) than at pH 6 (left). Scale bars = 100 µm.

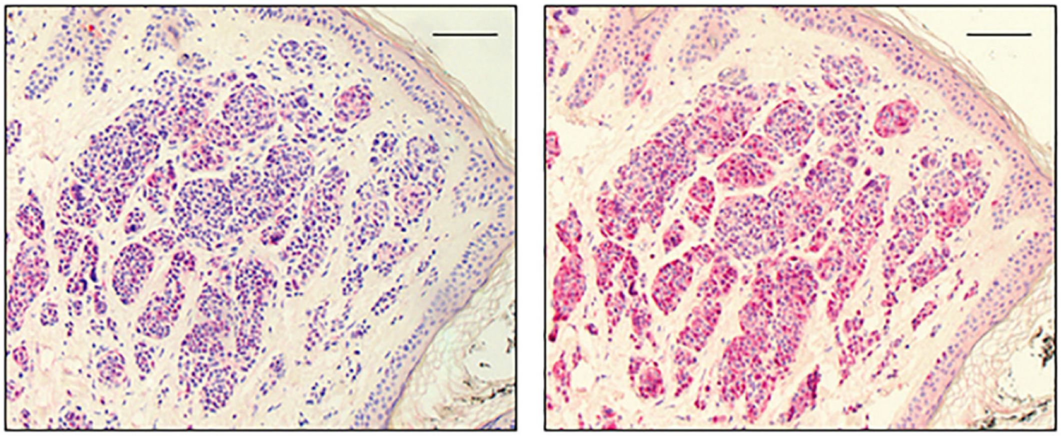

Supplementary Figure 4: IHC against p16 in nevus sections using an acidic or basic retrieval system. Immunostaining for p16 is clearly more intense, and more cells are positive after retrieval at pH 9 (right) than at pH 6 (left). Scale bars = 100 µm.

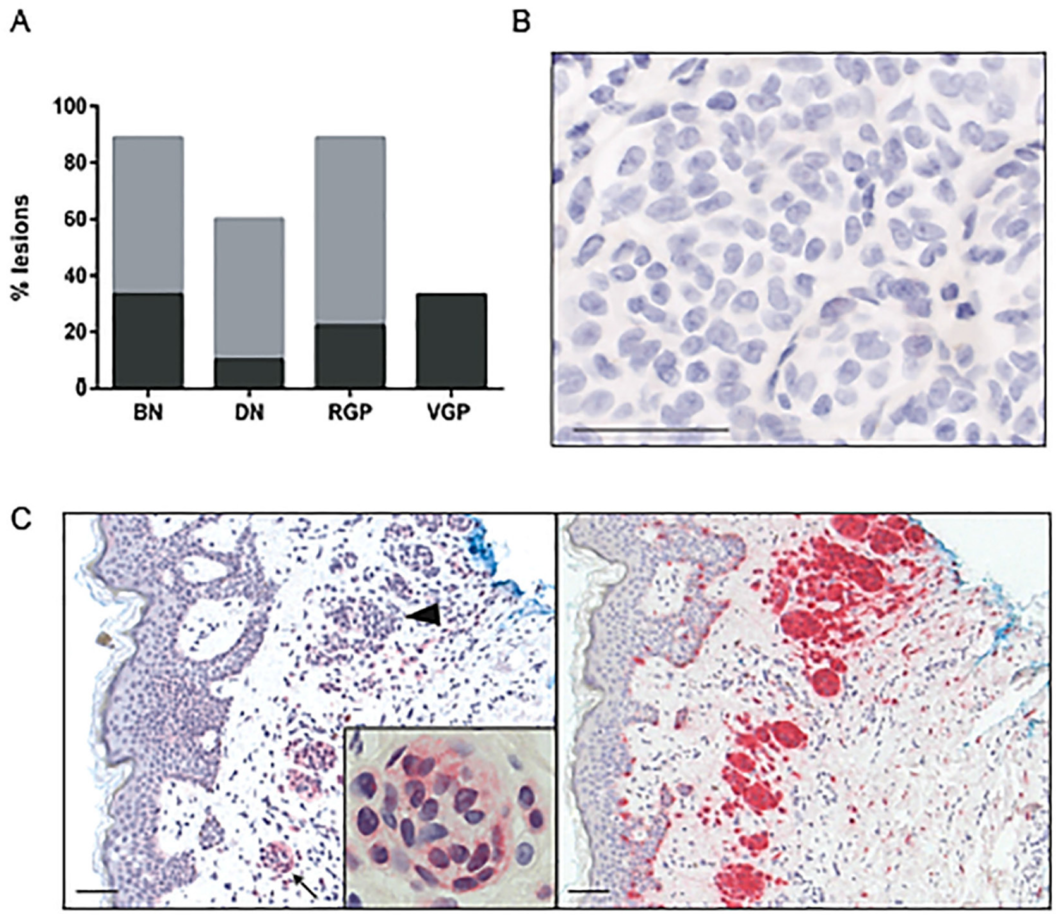

Supplementary Figure 5: p16 in melanoma progression: sensitive detection after pH 9 antigen-retrieval. (A) Nuclear p16 expression showing usual trend with melanoma progression (p=0.01, χ^2 test). Abbreviations and bar colour coding as in Figure 1. BN (n=10), DN (n=10), RGP (n=8) and VGP (n=9). **(B)** p16 negativity in a VGP melanoma. **(C)** p16 heterogeneity in a benign nevus, with nests negative (arrowhead) or positive for p16 (arrow, expanded in inset). Right: MART1 immunostain of a parallel section showing positions of nevus cells. Scale bars: 50 μm.

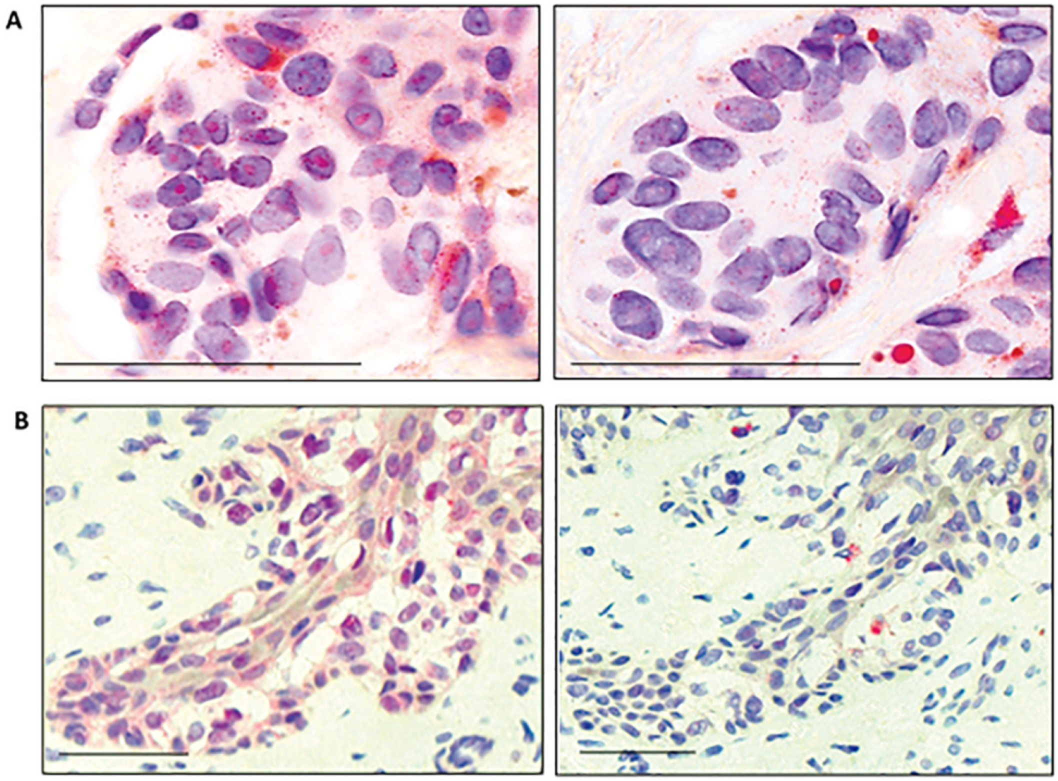

Supplementary Figure 6: IHC against TERT with or without a peptide block. Left images show **(A)** nucleolar TERT in a benign nevus and **(B)** non-nucleolar TERT in a VGP melanoma. Right images show negligible nuclear immunostaining after the antibody was blocked with 15 µg of epitope peptide. Some nonspecific staining remained after blocking, in cells tentatively identified as plasma cells, but this was always cytoplasmic. Scale bars = 50 µm.

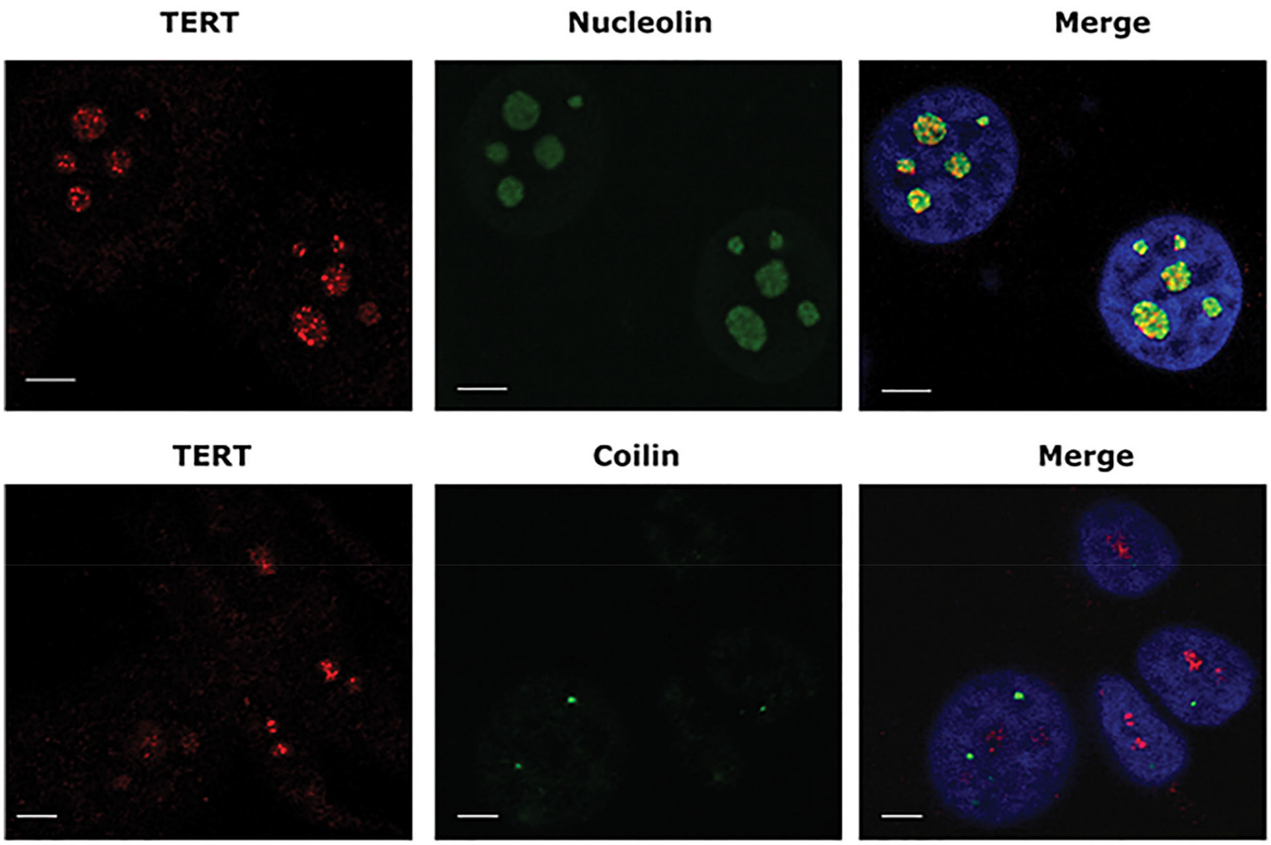

Supplementary Figure 7: TERT is still localized to nucleoli and not Cajal bodies in cultured A375P cells when a basic fixative is used. Nuclei are visualised with DAPI. Typical staining pattern. Scale bars = 5 µm. Other details as in Figure 5.
